# Rational selection of experimental readout and intervention sites for reducing uncertainties in computational model predictions

**DOI:** 10.1186/s12859-014-0436-5

**Published:** 2015-01-16

**Authors:** Robert J Flassig, Iryna Migal, Esther van der Zalm, Liisa Rihko-Struckmann, Kai Sundmacher

**Affiliations:** 1 Department Process Systems Engineering (PSE), Max Planck Institute for Dynamics of Complex Technical Systems, Sandtorstraße 1, Magdeburg, D-39106 Germany; 2 Department Process Systems Engineering, Otto von Guericke University Magdeburg, Universitätsplatz 2, Magdeburg, D-39106 Germany

**Keywords:** Computational modeling, Identifiability, Experimental design, Readout selection, Intervention site selection, Profile likelihood, Chlorophyll fluorescence induction

## Abstract

**Background:**

Understanding the dynamics of biological processes can substantially be supported by computational models in the form of nonlinear ordinary differential equations (ODE). Typically, this model class contains many unknown parameters, which are estimated from inadequate and noisy data. Depending on the ODE structure, predictions based on unmeasured states and associated parameters are highly uncertain, even undetermined. For given data, profile likelihood analysis has been proven to be one of the most practically relevant approaches for analyzing the identifiability of an ODE structure, and thus model predictions. In case of highly uncertain or non-identifiable parameters, rational experimental design based on various approaches has shown to significantly reduce parameter uncertainties with minimal amount of effort.

**Results:**

In this work we illustrate how to use profile likelihood samples for quantifying the individual contribution of parameter uncertainty to prediction uncertainty. For the uncertainty quantification we introduce the *profile likelihood sensitivity* (PLS) index. Additionally, for the case of several uncertain parameters, we introduce the PLS entropy to quantify individual contributions to the overall prediction uncertainty. We show how to use these two criteria as an experimental design objective for selecting new, informative readouts in combination with intervention site identification. The characteristics of the proposed multi-criterion objective are illustrated with an *in silico* example. We further illustrate how an existing practically non-identifiable model for the chlorophyll fluorescence induction in a photosynthetic organism, *D. salina*, can be rendered identifiable by additional experiments with new readouts.

**Conclusions:**

Having data and profile likelihood samples at hand, the here proposed uncertainty quantification based on prediction samples from the profile likelihood provides a simple way for determining individual contributions of parameter uncertainties to uncertainties in model predictions. The uncertainty quantification of specific model predictions allows identifying regions, where model predictions have to be considered with care. Such uncertain regions can be used for a rational experimental design to render initially highly uncertain model predictions into certainty. Finally, our uncertainty quantification directly accounts for parameter interdependencies and parameter sensitivities of the specific prediction.

**Electronic supplementary material:**

The online version of this article (doi:10.1186/s12859-014-0436-5) contains supplementary material, which is available to authorized users.

## Background

Advances in technology and biotechnology in particular allow us to look inside biological cells and observe dynamic processes occurring at the molecular level. Still, many of these processes can only partially be observed in experiments hampering the experimental exploration of interaction mechanisms. Here, a computational abstraction of the dynamic biochemical process in the form of an ordinary differential equation system (ODE) with unknown parameters can provide answers to the dynamics of unmeasured states that in turn give information on interaction mechanisms. Furthermore, model-based predictions and optimizations are possible. Such a model-based approach relies on the adequacy of the model, i.e. properly identified structure and parameter values. Often, the amount and quality of the experimental data is insufficient for complete model identification resulting into badly constrained or even non-identiafiable parameters, and thus uncertain dynamic model predictions. Although model predictions on observed data can be extremely certain and useful even in the presence of unidentifiable parameters (e.g. [1,2]), model predictions on unobserved (internal) model states that are related to these unidentifiable parameters can be highly uncertain. If model based predictions on internal states are of interest, experimental design can be used to rationally design new experiments with optimized content of information with respect to a specific model prediction. Several excellent publications have appeared over the last years, which focus on identification of computational models for biochemical systems by applying a variety of methodological optimal experimental design approaches, e.g. [3-8].

Profile likelihood estimation has been proven to be a valuable tool for parameter identifiability analysis [9]. Parameter identifiability analysis investigates whether a model parameter can be uniquely determined for the given data and input-output setting. For non-identifiable parameters, there exists an uncountable set of parameters, which yield the same model input-output behavior. As a result, predictions on internal states - states that are not directly observed in experiments - become highly uncertain [9]. An identifiability analysis can hint at a necessary re-design of an experimental input-output setup, a model re-parameterization or reduction to resolve non-identifiabilities [4].

In the following we briefly describe ODE modeling of biochemical processes, parameter estimation in ODE models and parameter identifiability analysis based on the profile likelihood. We then show how to use profile likelihood samples for quantifying individual contributions of parameter uncertainties to uncertainties in the model predictions by introducing the profile likelihood sensitivity index and profile likelihood sensitivity entropy. We further show, how to use this uncertainty quantification for experimental design by formulating the respective multi-criterion objective. The uncertainty quantification in combination with experimental design is illustrated with (i) an intuitive *in silico* example and (ii) a dynamic chlorophyll fluorescence induction model of the photosynthetic organism *D. salina*.

## Methods

### General model formulation

The dynamic evolution of the system’s states can be described by an ODE system in the form

(1) $$  \begin{aligned} \dot{x}(t)&=f\left(x(t, \theta_{x}), u(t), \theta_{x}\right) \\ y(t)&=g\left(x(t, \theta_{x}), \theta_{y}\right)+\epsilon, \end{aligned}  $$

where *x*(*t*,*θ*_*x*_) denotes the states of the system, *u*(*t*) indicates an external input function and *θ*_*x*_ a vector of dynamic system parameters. Experimental readouts *y*(*t*) are related to the model via the readout function *g*, which includes scaling and offset parameters *θ*_*y*_ and an additive white measurement noise model $\epsilon \propto N\left (0,\sigma _{\text {exp}}^{2}\right)$.

The temporal evolution of the states depends on the initial condition *x*(*t*=0), sufficiently smooth right hand side function *f*, external stimulus *u*(*t*) and kinetic system parameters *θ*_*x*_. Kinetic and readout parameters are combined into the parameter vector *θ*, including all parameters required to completely characterize the model

(2) $$ \theta=\left[\theta_{x}, \theta_{y}\right]^{\text{T}}.   $$

### Parameter estimation

Parameter estimates $\hat {\theta }$ of unknown values *θ* can be obtained by minimizing the residual sum of squares

(3) $$ \chi^{2}(\theta)=\sum\limits_{i=1}^{n} \left(y_{\text{exp}}(t_{i})-y_{\text{sim}}(t_{i})\right)^{2} /\sigma^{2}_{\text{exp}}(t_{i}).   $$

Here, *y*_exp_(*t*_*i*_) denotes measured data at time points *t*_*i*_ (*i*=1…*n*, with *n* number of time points) and *y*_sim_(*t*_*i*_) indicates the model output for time points *t*_*i*_. For the assumed measurement noise model and likelihood *L* we have *χ* ^2^∝−2 log*L* and $\hat {\theta }$ corresponds to the maximum likelihood estimate (MLE). In the following we thus use *χ* ^2^ as a placeholder for the likelihood.

### Profile likelihood

The profile likelihood of a parameter represents a constrained projection of the likelihood in the typically high dimensional parameter space. Following [9], the profile likelihood of a parameter *θ*_*i*_ is given by

(4) $$ \chi^{2}_{\text{PL}}(\theta_{i})={\underset{\theta_{j\neq i}}{\text{min}}}\chi^{2}(\theta),  $$

which represents a function in *θ*_*i*_ of least increase in the likelihood. The least increase is achieved by adjusting *θ*_*j*_,*j*=1…*n*_*θ*_∖*i* accordingly.

Further, profile likelihood-based confidence regions CR can be derived via

(5) $$ \text{CR}=\left\{\theta | \chi_{\text{PL}}^{2}(\theta)-\chi_{\text{PL}}^{2}(\hat{\theta}) < \delta_{\alpha}\right\},   $$

with *δ*_*α*_ being the *α* quantile of the *χ* ^2^ distribution with *d* *f*=1 (pointwise) or *d* *f*=*n*_*θ*_ (simultaneous) degrees of freedom [9]. A confidence interval of parameter *θ*_*i*_ is simply given by the borders of CR.

### Uncertainty quantification based on profile likelihood sensitivity indices

There exist many advanced methods to analyze and quantify uncertainty propagation in ODE models. In biochemical systems modeling, efficient sampling strategies, including MCMC, profile likelihood and sigma points have been successfully applied to uncertainty analysis in real systems [2,10,11]. These methodological approaches are - in contrast to approaches based on classical Fisher Information (FI) - especially effective in cases of highly nonlinear models, as the nonlinearity is more adequately accounted for. This also holds for the model-based experimental design: Our presented approach is a sample based approach, whereas FI relies on curvature information of the likelihood. As has been shown by several authors, including [9,12], FI may not be well suited for non-linear models. In contrast, the profile likelihood approach accounts for a possible non-linear character of the model. Further, FI-based approaches operate on the covariance matrix in the parameter space and can be given a geometrical interpretation: FI criteria measure the shape and orientation of an *n*_*θ*_ dimensional ellipsoid (to be more specific, the inverse of FI is used). By optimizing such FI-based criteria (e.g. A-, D-, E-optimality), one tries to reduce and distribute uncertainties and their correlations in the parameter space. In the case of non-linear models, this does not guarantee that model-based predictions other than parameter values become more constrained. Our approach (PLS index and entropy) differs in this that it operates in the prediction space (which can also include predictions on parameter values). In this way, our approach is more general. Additionally, profile likelihood samples are readily available once a practically identifiability analysis based on the profile likelihoods (one of the practically most relevant approaches in systems biology) has been performed by the modeler.

In the following we build on results of [4,9], who already proposed to use the set of parameters along the profile likelihood for analyzing the impact of parameter uncertainties on model states or more generally model predictions *p*. Notably, unidentifiable or poorly constrained parameters can induce large variations in unmeasured model states and corresponding predictions. We thus define a measure of individual uncertainty impact for a parameter *θ*_*i*_ on a dynamic model prediction $p(t_{k})\in \mathbb {R}$ over a finite time horizon as

(6) $$ s_{i}(t_{k})=\left(\frac{{\text{max}}\left(\left\{p_{i}(t_{k})\right\}\right)-{\text{min}}\left(\left\{p_{i}(t_{k})\right\}\right)}{\langle \hat{p}(t)\rangle_{t}}\right)^{2},   $$

which we refer to as the *profile likelihood sensitivity* index (PLS index) of parameter *θ*_*i*_ for prediction *p* at time *t*_*k*_. Note that expressions max/min({·}) define the maximum and minimum (=extremes) over the set $\{\cdot \}\subset \mathcal {P}_{i}$, which contains model predictions *p*_*i*_(*t*_*k*_) sampled along the profile likelihood of parameter *θ*_*i*_∈CR_*i*_. In the case where *p*_*i*_(*t*_*k*_)≡*x*(*t*_*k*_,*θ*_*i*_) and finite confidence interval of parameter *θ*_*i*_, max/min({·}) approximate the confidence band around the MLE state trajectory *x* ^MLE^(*t*). This also holds for an arbitrary prediction *p*. If model parameters are unidentifiable, their respective confidence interval is unbounded. In this case, we suggest sampling a reasonable large range along the profile likelihood (say 3 orders of magnitudes) around the MLE of the unidentifiable parameters. In this way, the impact of unidentifiable parameters on so far unobserved predictions *p*_*i*_(*t*_*k*_) is revealed via *s*_*i*_(*t*_*k*_). The denominator $\langle \hat {p}(t)\rangle _{t}$ in Eq. (6) represents the time average of the prediction at the MLE of the parameters.

Having several uncertain model parameters, an overall uncertainty quantification for a model prediction is given by

(7) $$ s_{\text{tot}}=\sum_{i=1}^{n_{\theta}}\sum_{k=1}^{n_{t}} s_{i}(t_{k}).   $$

To measure individual parameter uncertainty contributions to *s*_tot_ we suggest to use

(8) $$ J_{tot}=\sum_{k=1}^{n_{t}} J_{k},   $$

which is based on Shannon’s entropy given by

(9) $$ J_{k}=\sum_{i=1}^{n_{\theta}}-\tilde{s}_{i}(t_{k})\log\left(\tilde{s}_{i}(t_{k})\right)\\  $$

with

(10) $$ \tilde{s}_{i}(t)=\frac{s_{i}(t_{k})}{\sum_{i=1}^{n_{\theta}}s_{i}(t_{k})}.  $$

Shannon’s entropy measures how homogenous PLS indices *s*_*i*_(*t*_*k*_) contribute to *s*_tot_.

### Experimental design to reduce prediction uncertainties

PLS indices can be used to identify highly uncertain predictions and thus provide guidance in designing new, informative experiments: An optimal experimental design (OED) that maximizing the PLS index for an individual parameter corresponds to an experimental region, where the uncertainty of this parameter induces maximal uncertainty in the model prediction. Therefore, if one is interested in reducing the uncertainty of a specific prediction *p*(*t*_*k*_) by an additional experiment, one would simply select an experimental design that maximizes the PLS index of *p*(*t*_*k*_). Note however, that if one wants to reduce the overall uncertainty of a model prediction as a result of several uncertain model parameters it is not sufficient to identify an experimental region that maximizes Eq. (7). Similar to A-, D- or E-optimality based on FI, one has to trade off maximal *s*_tot_ and more or less equal contributions to *s*_tot_ by all uncertain parameters. Here, the measure in Eq. (8) should be maximized aiming at equal contributions of *s*_*i*_(*t*_*k*_) and corresponding parameter uncertainties. Such a design should produce homogenous parameter information in the data with respect to the prediction goal of the model.

An often targeted prediction goal is the analysis of unmeasured model states, thus *p*_*ij*_(*t*_*k*_)≡*x*_*j*_(*t*_*k*_,*θ*_*i*_) with *j*∈{unmeasured states}. Two design scenarios can be distinguished: if one is to choose a set of new, additional readouts from the set {unmeasured states}, one would select states *x*_*j*_ that maximize the objective *O*=[*s*_*j*,tot_ *J*_*j*,*tot*_]^T^. If one cannot select a new readout, other design variables as for instant intervention sites (e.g. inhibition of states or associated reactions), stimulus profiles or selection of measurement time points can also be used to optimize the objective *O* for a given readout setup. Both design scenarios may also be combined. In the *Results and discussion* section we illustrate how to select additional readouts and/or inhibition sites.

### Cultivation of Dunaliella salina

In this part the experimental procedures are described that have been used to obtain the data for the photosynthetic application. The *Dunaliella salina* strain CCAP19/18 [13] was used, which has been ordered from CCAP (www.ccap.ac.uk). Bacteria in the medium were killed by 100 *μ*M chloramphenicol; other contaminating organisms were not present (PDA tests for fungal contamination; light microscopy at 1000x with oil immersion, Zeiss Axio Image). Medium composition was used as described in [14], but modified by addition of 40 mM Hepes pH 7.5. 1-3 ml was inoculated in 100 ml sterile medium. Cultures were grown in a shaking incubator (Infors HT) at 100 rpm at 16/8 h light /dark cycle at 26°C and 3.5*%* CO _2_. FL tubes Gro-Lux 15 W Fluorescent Lamps Sylvania type F15W /*GRO*/ were used as light source; intensity was 30-60 *μ* *Em* ^−2^ *s* ^−1^. For chlorophyll fluorescence (Chl F) measurements 7-14 d old cultures were used.

### Chlorophyll fluorescence measurements

The DUAL-PAM-100 (Walz, Effeltrich, Germany) using the DUAL-E emitter (actinic light = 620 nm; measuring light = 460 nm) and DUAL-DB detector was used for Chl F measurements. A cell density of 10^7^ ml ^−1^ was taken (adjusted with help of cell counting with the Cellometer Auto T4 Plus, PEQLAB). Measuring light frequency and intensity were adjusted to 500 Hz, and 3 *μ* *Em* ^−2^ *s* ^−1^, respectively. Before performing fluorescence measurements, samples were kept in the dark for 10 min. Temperature during pre-incubation and measurement was kept at 23±0.5°C, and cell suspensions were stirred to prevent cell sedimentation. A light pulse of 166 *μ* *Em* ^−2^ *s* ^−1^ and duration of 1 s was applied to the cell suspension; 6 replicate measurements were performed at a sampling of *Δ* *t*=10^−4^ s. A light intensity of 166 *μ* *E* *m* ^−2^ *s* ^−1^ was selected. This intensity produced the typical chlorophyll fluorescence induction curve.

## Results and discussion

### In silico example

**Figure 1:**
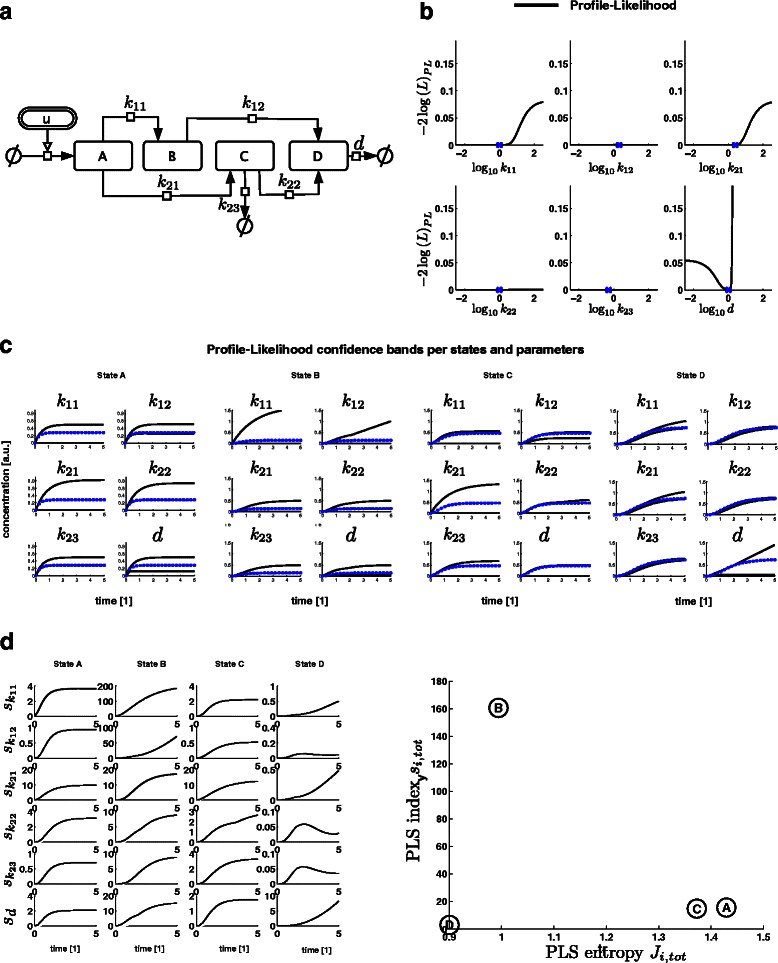
***In silico*** **example for profile likelihood sensitivity based experimental design.** **(a)** Interaction structure of the *in silico* model. **(b)** Profile likelihood for the 6 model parameters given *in silico* time-course data of *y*= D. The blue cross indicates the MLE of *θ*_*i*_. The 95% point-wise significance is at $\chi ^{2}_{\text {crit}}=3.68$. **(c)** Simulated extreme state trajectories along the profile likelihood for each parameter. **(d)** On the left side the temporal evolution of the PLS index *s*_*ij*_(*t*) is shown for each parameter (black) with *j*={*A*,*B*,*C*,*D*}. The corresponding criterion space derived from *s*_*ij*_(*t*) is shown on the right.

To illustrate PLS index- and entropy-based experimental design, we here consider a simple *in silico* example. Figure 1(a) illustrates the interaction network of 4 species A, B, C and D, from which we derived an ODE system using mass action kinetics (s. Supplementary). An initial data set consisting of time-course data of state D is given, so are the parameter estimates of the 6 kinetic parameters, which are assumed to be unknown. All initial conditions are set to zero and assumed to be known. In Figure 1(b) profile likelihood are calculated on the basis of this initial data set. The profile likelihood reveals that all parameters are non-identifiable. Only the upper bound of parameter *d* is constrained, implying that the maximal degradation rate of D is limited. This result could be expected from the model structure. Two parallel pathways that activate the readout state D in combination with an additional sink provide too much flexibility, and thus arbitrarily sets of parameters that still allow the readout trajectory of state D to fit the measured data. Therefore, predictions on unmeasured states are highly uncertain as is illustrated by the extreme trajectories of each parameter derived from the simulated samples along the profile likelihood (Figure 1(c)). The corresponding PLS indices are shown in Figure 1(d). In the next step we illustrate, how to select new readouts in order to reduce the uncertainty in the model state predictions.

#### Optimal readout selection

**Figure 2:**
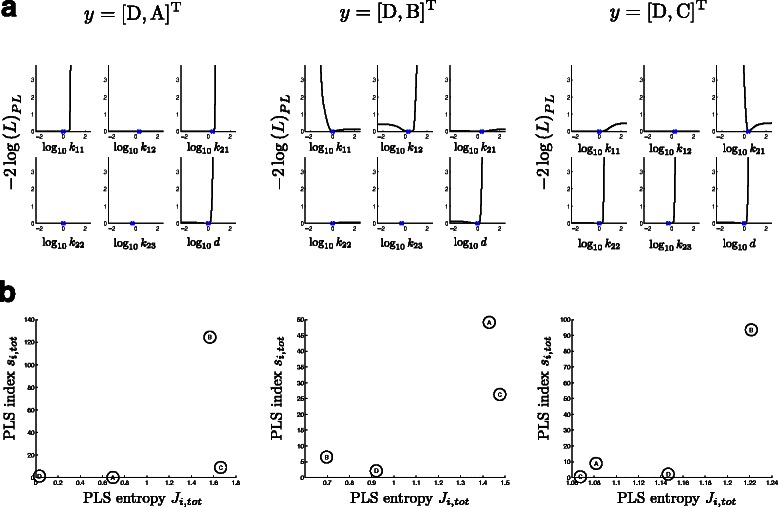
**Rational readout selection via profile likelihood sensitivity indices.** **(a)** Profile likelihoods for indicated readout setups. **(b)** PLS entropy vs. PLS index derived from the extreme state trajectories provide the basis for a new readout selection.

In Figure 1(d), right side, the criterion space shows *s*_*i*,tot_ and *J*_*i*,tot_ based on the profile likelihood samples derived for the initial data set (time-course data for state D only). It seems that the uncertainty in the prediction of state B is largest (highest PLS index), however induced by fewer parameters compared to A or C, which have larger PLS entropies compared to B. Since D has been already used for parameter estimation, it is not as much uncertain, indicated by the smallest PLS index. In Figure 2(a) the profile likelihoods are recalculated for an additional readout, i.e. **y**=[D, A]^T^ or [D, B]^T^ or [D, C]^T^. The corresponding uncertainty in the state predictions are illustrated by the extremes of the profile likelihood samples in Figure 2(b). Qualitatively, overall uncertainties are reduced, whereas data from [ D, A]^T^ allow constraining upper bounds. In line with the derived PLS indices and entropies, data from [D, B]^T^, [D, C]^T^ are more informative, since also lower bounds of *k*_11_ or *k*_21_ can be given. Theses two parameters govern the activation of B or C, respectively. Regarding uncertainties in unmeasured state predictions, readout setups **y**=[D, A]^T^ or **y**=[D, C]^T^ shift uncertainties mostly to state B, whereas readout setup **y**=[D, B]^T^ distributes the remaining uncertainty onto states A and C. This observation motivates an anticipatory design strategy (e.g. [15]) to not only identify the most uncertain state prediction but to identify an unmeasured state as a new readout that reduces the overall uncertainties in all unmeasured states: perform *in silico* experiments for potential new readouts and evaluate the PLS index. Then, select the readout which reduces the PLS index over the set of unmeasured states.

For any readout setup, none of the parameters are identifiable, although some upper lower parameter bounds can be derived, see Table 1. Based on the profile likelihood samples of the readout setups in Figure 2(a),(b) the criterion space was computed (Figure 2(c)), for identifying an additional (third) readout. Then again, profile likelihoods have been calculated for the readout setups [D, A B]^T^, [D, A C]^T^, [D, B C]^T^ and also [D A, B, C, D]^T^. The resulting identifiabilities and confidence bounds are given in Table 2. In line what is predicted by the criterion space in Figure 2(c), B is an important readout if not already measured: Measuring any of the two 3-state combinations [D, A B]^T^, [D, B C]^T^ where B is always included is as informative as a 4 state readout setup for the given input design, sampling rate and noise setting. Finally note that even though all states have been measured, data are not sufficient for a complete identification of the model.

**Table 1:** **Profile likelihood based identifiability analysis and confidence intervals of the** ***in silico*** **model example in log-space**

**Parameter**	$\hat {\theta }_{i}$	***y*** **= D**			***y*** **=[** ***D*** **,** ***A*** **]** ^**T**^			***y*** **= [D, B]** ^**T**^			***y*** **= [D, C]** ^**T**^		
		**Identifiability**	**Lower CI**	**Upper CI**	**Identifiability**	**Lower CI**	**Upper CI**	**Identifiability**	**Lower CI**	**Upper CI**	**Identifiability**	**Lower CI**	**Upper CI**
log10*k*_11_	0.043	non-identifiable	−*∞*	*∞*	non-identifiable	−*∞*	0.65	non-identifiable	−1.08	*∞*	non-identifiable	−*∞*	*∞*
log10*k*_12_	0.301	non-identifiable	−*∞*	*∞*	non-identifiable	−*∞*	*∞*	non-identifiable	−*∞*	1.07	non-identifiable	−*∞*	*∞*
log10*k*_21_	0.398	non-identifiable	−*∞*	*∞*	non-identifiable	−*∞*	0.65	non-identifiable	−*∞*	*∞*	non-identifiable	−0.03	*∞*
log10*k*_22_	0.004	non-identifiable	−*∞*	*∞*	non-identifiable	−*∞*	*∞*	non-identifiable	−*∞*	*∞*	non-identifiable	−*∞*	0.45
log10*k*_23_	−0.301	non-identifiable	−*∞*	*∞*	non-identifiable	−*∞*	*∞*	non-identifiable	−*∞*	*∞*	non-identifiable	−*∞*	0.33
log10*d*	0.004	non-identifiable	−*∞*	0.42	non-identifiable	−*∞*	0.42	non-identifiable	−*∞*	0.42	non-identifiable	−*∞*	0.4

This table illustrates the potential impact of one additional readout to the initial setup *y* = D.

**Table 2:** **Profile likelihood based identifiability analysis and confidence intervals of the** ***in silico*** **model example in log-space**

**Parameter**	${\hat {\theta }_{i}}$	***y*** **= [D, A B]** ^**T**^			**y= [D, A C]** ^**T**^			***y*** **=[D, B C]** ^**T**^			***y*** **=[D, A B C]** ^**T**^		
		**Identifiability**	**Lower CI**	**Upper CI**	**Identifiability**	**Lower CI**	**Upper CI**	**Identifiability**	**Lower CI**	**Upper CI**	**Identifiability**	**Lower CI**	**Upper CI**
log10*k*_11_	0.043	identifiable	−0.72	0.44	non-identifiable	−*∞*	0.41	identifiable	−0.86	1.17	identifiable	−0.70	0.36
log10*k*_12_	0.301	non-identifiable	−*∞*	0.86	non-identifiable	−*∞*	*∞*	non-identifiable	−*∞*	0.82	non-identifiable	−*∞*	0.81
log10*k*_21_	0.398	identifiable	−0.16	0.60	identifiable	0.07	0.63	identifiable	−0.01	1.45	identifiable	0.12	0.59
log10*k*_22_	0.004	non-identifiable	−*∞*	3	non-identifiable	−*∞*	0.44	non-identifiable	−*∞*	0.39	non-identifiable	−*∞*	0.39
log10*k*_23_	−0.301	non-identifiable	−*∞*	*∞*	non-identifiable	−*∞*	0.31	non-identifiable	−*∞*	0.29	non-identifiable	−*∞*	0.23
log10*d*	0.004	non-identifiable	−*∞*	0.42	non-identifiable	−*∞*	0.40	non-identifiable	−*∞*	0.39	non-identifiable	−*∞*	0.37

This table illustrates the potential impact of additional readouts to the initial setup *y* = D.

#### Optimal readout and inhibition site selection

**Figure 3:**
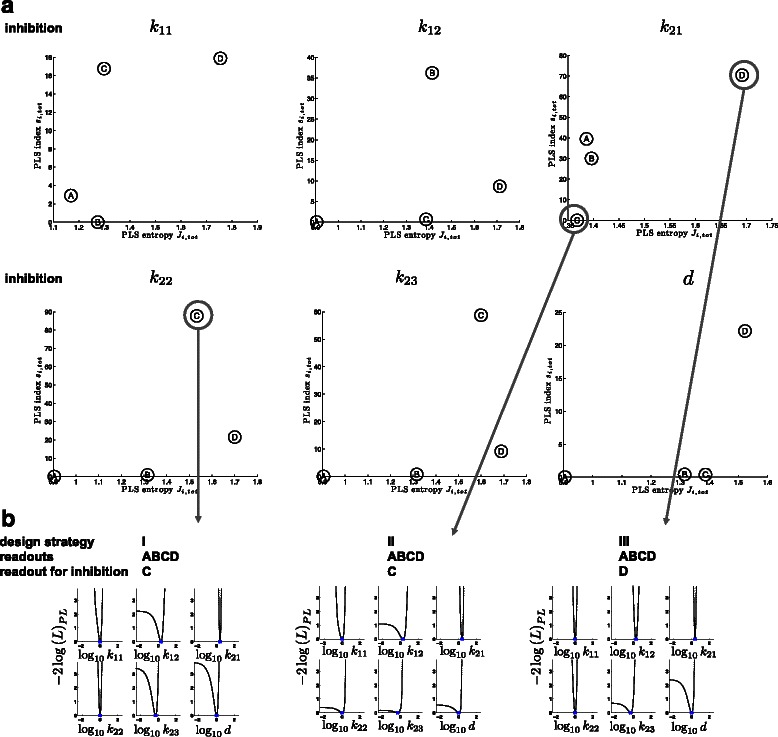
**Experimental design: inhibition and readout selection for best parameter estimation.** **(a)** Criterion space for all 6 possible single inhibition scenarios. An inhibition of a specific reaction is simulated by reducing the corresponding kinetic parameters by a factor of 10^−4^. **(b)** Profile likelihoods for different readout and inhibition scenarios (indicated by the gray arrows).

Here we investigate the impact of inhibiting a certain reaction. We model the inhibition of a certain reaction by reducing the corresponding parameter value by the factor of 10^−4^, which may seem arbitrary, but is used to illustrate the concept of inhibition selection. For a real life application one would have to consider the efficiency of a specific inhibitor. Starting from initial measurements in [A,B,C,D]^T^, we now wish to identify one inhibition that maximizes parameter identifiability. In Figure 3(a) the criterion space is shown for all 6 inhibition scenarios. Inhibition of the reaction associated to parameter *k*_21_ in combination with measurement of D (design strategy I) or inhibition of *k*_22_ in combination with measuring C (design strategy III) seem good experimental designs. A suboptimal example is design strategy II, i.e. measuring C and inhibiting *k*_21_, which is indicated by the small PLS index. In Figure 3(b) we show the corresponding profile likelihoods. Consistent with the prediction in the criterion space strategies I and III improve the identifiability of the parameters (Table 3). Still, not all parameters can be identified. The benefit of the entropy measure can also be seen (Figure 3(b) and Table 3): design strategy III is based on larger PLS than strategy I for the specific readouts, however, contributions of individual PLSs are larger for strategy I, which results into better information distribution along the parameters, as is indicated by the shapes of the profile likelihood but also by the ranges of the confidence intervals and number of identifiable parameters.

**Table 3:** **Profile likelihood based identifiability analysis and confidence intervals of the** ***in silico*** **model example in log-space**

**Parameter**	$\hat {\theta }_{i}$	$y=\left [\text {A B C D, C}_{k_{22}}\right ]^{\text {T}}$			$y=\left [\text {A B C D, C}_{k_{21}}\right ]^{\text {T}}$			$y=\left [\text {A B C D, D}_{k_{21}}\right ]^{\text {T}}$		
		**Identifiability**	**Lower CI**	**Upper CI**	**Identifiability**	**Lower CI**	**Upper CI**	**Identifiability**	**Lower CI**	**Upper CI**
log10*k*_11_	0.043	identifiable	−0.62	0.29	identifiable	−0.70	0.36	identifiable	−0.25	0.23
log10*k*_12_	0.301	non-identifiable	−*∞*	0.73	non-identifiable	−*∞*	0.81	identifiable	−0.02	0.63
log10*k*_21_	0.398	identifiable	0.24	0.55	identifiable	0.12	0.59	identifiable	0.23	0.54
log10*k*_22_	0.004	identifiable	−0.23	0.21	non-identifiable	−*∞*	0.39	identifiable	−0.33	0.24
log10*k*_23_	−0.301	non-identifiable	−*∞*	0.02	non-identifiable	−*∞*	0.23	non-identifiable	−*∞*	0.17
log10*d*	0.004	non-identifiable	−*∞*	0.28	non-identifiable	−*∞*	0.37	non-identifiable	−*∞*	0.32

This table illustrates the effect of an additional inhibition and readout selection. The additional readout and inhibition is indicated by the 5th letter and subscripted parameter, which corresponds to the inhibited reaction.

### Photosynthetic organism *D. salina*

#### Model description

**Figure 4:**
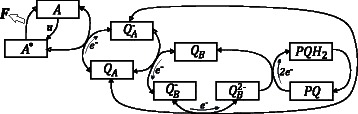
**Model interaction structure of the fluorescence induction model for** ***D. salina*** **.** Black arrows indicate the forward reactions, while white (not filled) arrows denote backward reactions. *F* is the dissipated chlorophyll fluorescence, **u** is the intensity of the excitation light. *A* denotes the unexcited antennae, *A* ^∗^ is the number of excited antennae. *Q*_*A*_ and *Q*_*B*_ are the first and the second quinone receptors respectively and $Q_{A}^{-}$, $Q_{B}^{-}$ and $Q_{B}^{2-}$ represent their oxidized states. *PQ* is the plastoquinon pool and *P* *Q* *H*_2_ represents its protonated form.

The dynamic process of chlorophyll fluorescence induction of photosynthesis in green plants was chosen as a real life application. The timescale of this dynamic process is in the order of milli seconds. The model derived in [16] was used to describe the dynamics of the chlorophyll fluorescence induction. It consists of four reversible and two irreversible electrochemical reactions. A model scheme is given in Figure 4. The following system of ordinary differential equations describes the dynamical behavior of the model [16]

(11) $$ {\fontsize{8}{12}\begin{aligned} \dot{x}_{1}&=k_{1}u\left(A_{0}-x_{1}\right)-k_{2}x_{1}-k_{3}x_{1}\left(1-x_{2}\right)+k_{4}x_{2}\left(A_{0}-x_{1}\right) \\[-1pt] \dot{x}_{2}&=k_{3}x_{1}\left(1-x_{2}\right)-k_{4}x_{2}\left(A_{0}-x_{1}\right)-k_{5}x_{2}\left(r_{2}-x_{3}-x_{4}\right)\\[-1pt] & \quad +k_{6}x_{3}\left(1-x_{2}\right)-k_{7}x-2x_{3}+k_{8}x_{4}\left(1-x_{2}\right) \\[-1pt] \dot{x}_{3}&=k_{5}x_{2}\left(r_{2}-x_{3}-x_{4}\right)-k_{6}x_{3}\left(1-x_{2}\right)-k_{7}x_{2}x_{3}+k_{8}x_{4}\left(1-x_{2}\right)\\[-1pt] \dot{x}_{4}&=k_{7}x_{2}x_{3}-k_{8}x_{4}\left(1-x_{2}\right)-k_{9}x_{4}x_{5} \\[-1pt] \dot{x}_{5}&=-k_{9}x_{4}x_{5}+k_{10}\left({PQ}_{0}-x_{5}\right). \end{aligned}}  $$

The *x*_1_…*x*_5_ denote probabilities of states *A* ^∗^, $Q_{A}^{-}$, $Q_{B}^{-}$, $Q_{B}^{2-}$ and *PQ* (in this order). Corresponding concentrations can be derived by multiplying *x*_*i*_ with the reaction center concentration in a sample. *A*_0_ is the size of the antenna pool (number of antennae) and *P* *Q*_0_ is the size of *PQ*-pool per reaction center. The intensity of the excitation light is indicated with *u* and *k*_1_…*k*_10_ are kinetic parameters. The readout is given by

(12) $$ F=G k_{2} x_{1},   $$

where *G* is the overall gain or scaling coefficient that captures influences of sample size, concentration of the reactions centers or instrumental amplification. A vector of the initial state values is defined as follows:

$$x_{0}=\left[0\; 0\; 0\; 0\; {PQ}_{0}\right] $$

According to [17] there are 290 antennae in one antenna complex and thus we fixed *A*_0_=290. A total number of 13 unknown model parameters have to be estimated from the data.

#### Parameter estimation and identifiability

The parameters were estimated from fluorescence data of *Dunaliella salina* using PAM-fluorimeter under conditions specified in the *Chlorophyll fluorescence measruements* section. Following [18], data replicates were normalized according to

(13) $$ F_{n}=\frac{F-F_{0}}{F_{m}-F_{0}},   $$

in order to allow estimating sample mean and standard deviations from the replicates. *F* denotes the fluorescence value at a given time point *t*, *F*_0_ is the ground fluorescence at *t*=0 and *F*_*m*_ is the maximal measured fluorescence.

**Figure 5:**
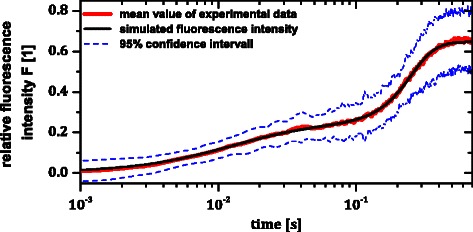
**Comparison of the results of model simulation with experimental data.** Experimental data were measured at 166*μ* *E* *m* ^−2^ *s* ^−1^. Simulation results (black line) are plotted versus the experimental data (red line). Blue dashed lines indicate the 95*%* confidence interval of the experimental data. The MLE corresponds to *χ* ^2^=378.5.

The MLE of the parameters are given in Table 4. Simulated and experimental data are plotted in Figure 5 on logarithmic time scale to represent all significant phases of the fluorescence induction. As shown in Figure 5, model simulation perfectly fits the experimental data. However, profile likelihood analysis revealed that one model parameters is non-identifiable (see below), rendering the analysis of internal state dynamics - as for instance performed in [16], who also only used fluorescence data - questionable.

**Table 4:** **Parameter identifiability of the** ***D. salina*** **model and confidence intervals based on original data and** ***in silico*** **experiments in log-space**

**Parameter**	$\boldsymbol {\hat {\theta }_{i}}$	**Original data:** ***y=Gk***_***2***_ ***x***_***1***_			**Add** ***in silico*** ***y*** **=** ***x***_**3**_			**Add** ***in silico*** ***y*** **=** ***x***_**5**_		
		**Identifiability**	**Lower CI**	**Upper CI**	**Identifiability**	**Lower CI**	**Upper CI**	**Identifiability**	**Lower CI**	**Upper CI**
log10*k*_1_	-1.9507	identifiable	-1.9526	-1.9481	identifiable	-1.9507	-1.9507	identifiable	-1.9512	-1.9499
log10*k*_2_	1.2603	identifiable	1.2603	1.2663	identifiable	1.2624	1.2641	identifiable	1.2626	1.2648
log10*k*_3_	3.3192	identifiable	3.3160	3.3251	identifiable	3.3173	3.320	identifiable	3.3183	3.3202
log10*k*_4_	2.0231	identifiable	2.0192	2.0269	identifiable	2.0220	2.0240	identifiable	2.0218	2.0239
log10*k*_5_	4.5278	identifiable	4.5214	4.5351	identifiable	4.5278	4.5278	identifiable	4.5264	4.5296
log10*k*_6_	5.5047	identifiable	5.4963	5.5128	identifiable	5.5030	5.5056	identifiable	5.5030	5.5062
log10*k*_7_	4.6765	identifiable	4.6683	4.6841	identifiable	4.6742	4.6774	identifiable	4.6752	4.6786
log10*k*_8_	2.8452	identifiable	2.8222	2.8602	identifiable	2.8439	2.8464	identifiable	2.8406	2.8487
log10*k*_9_	1.5753	identifiable	1.5697	1.5839	identifiable	1.5741	1.5759	identifiable	1.5749	1.5757
log10*k*_10_	0.1611	non-identifiable	−*∞*	0.5602	identifiable	0.1611	0.1611	identifiable	0.1606	0.1616
log10*P* *Q*_0_	1.4930	identifiable	1.4907	1.4947	identifiable	1.4922	1.4935	identifiable	1.4927	1.4931
log10*r*_2_	-0.3069	identifiable	-0.3106	-0.3025	identifiable	-0.3069	-0.3069	identifiable	-0.3073	-0.3065
log10*G*	-2.8061	identifiable	-2.8067	-2.8036	identifiable	-2.8061	-2.8061	identifiable	-2.8068	-2.8055

#### Optimal experimental design for *D. salina*: Readout selection

**Figure 6:**
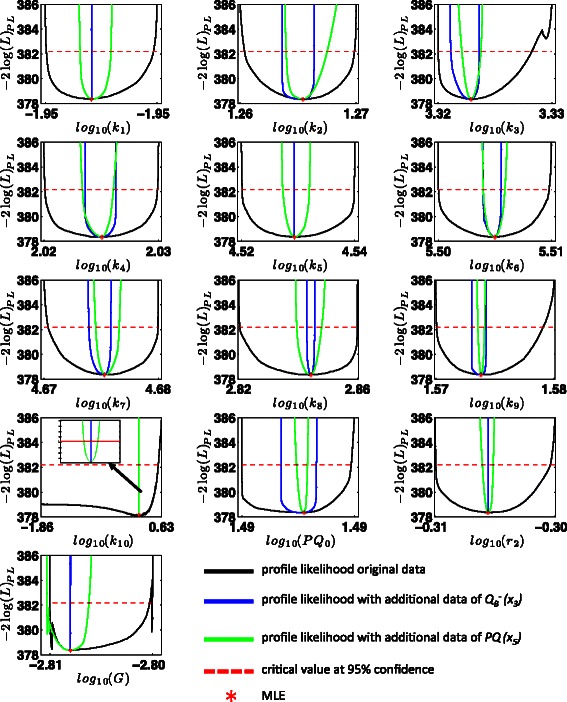
**Overview of the profile likelihoods of all parameters.** Solid lines indicate profile likelihood versus parameter on a *l* *o* *g*_10_-scale.

The profile likelihood for the initial data suggests, that 12 out of 13 model parameters are identifiable. Only parameter *k*_10_ has not lower bound (see Figure 6 and Table 4). Based on the estimated profile likelihoods, we calculated PLS indices and entropies to identify additional informative readout candidates for parameter *k*_10_.

As it turned out, states $Q_{B}^{-}$ and $Q_{B}^{2-}$ seem good candidates as additional readouts, having large PLS indices (see Additional file 1: Figure S5 for the criterion space), whereas $Q_{A}^{-}$ has the largest PLS entropy. *PQ* seems to equally trade-off the PLS index vs. PLS entropy. Since it was not possible to measure these internal states directly, *in silico* values for $Q_{B}^{-}$ and *PQ* were generated for the MLE parameter set to test their suitability as additional readouts. The *in silico* data were then used to perform identifiability analysis with the profile likelihood approach. The results are presented in Table 4, the corresponding profile likelihoods can be found in Figure 6. The additional *in silico* data for $Q_{B}^{-}$ or *PQ* allow identifying all parameters at *α*=0.05 (s. Table 4). Then, it would be possible to perform a conclusive, model-based analysis of the fluorescence induction in *D. salina* including all unmeasured states.

## Conclusions

In this work we illustrate how to analyze and quantify uncertainty propagation in ODE models based on profile likelihood samples. We introduce the profile likelihood sensitivity index, which reflects the individual contribution of an uncertain model parameter to a model prediction. In the case of several parameters, parameter interdependencies are - by definition of the profile likelihood - account for, and the sum of profile likelihood sensitivity indices can be used to quantify the overall effect. However, individual parameter uncertainty contributions are not clear. Here we propose to use Shannon’s entropy on the individual profile likelihood sensitivity indices as an additional measure. The PLS entropy describes the amount of uncertainty contributed by each uncertain parameter to the overall PLS index. In this way, PLS entropy can be used to look for homogenous uncertainty contribution. We further describe in a general way, how profile likelihood sensitivity index and entropy can be used to identify experimental regions, where one has to collect data in order to reduce prediction uncertainties. Such an approach is especially valuable in large biochemical networks, where intuitive analysis is hampered by the complexity of the system. Additionally, PLS index and entropy provide information on prediction domains, where one has to consider model-based predictions with care.

We applied the concept of PLS indices and entropies to an intuitive *in silico* example to illustrate how one can rationally select additional readout and/or intervention sites in order to reduce prediction uncertainties. Finally, using the chlorophyll fluorescence induction of *D. salina* as a true life case, we illustrate how an initially non-identifiable model can potentially be rendered identifiable by selection additional readout signals.

## Availability of supporting data

The supplementary material contains the ODE system of the *in silico* example, further details on the fluorescence induction model of *D. salina* (comparison of profile likelihood and classical sensitivity analysis including time course of PLS indices, prediction samples and criterion space for readout selection). We further provide MATLAB code for the *in silico* example to explore the presented design approach and also data and MATLAB code for the chlorophyll fluorescence induction model (Additional file 2). Further material and support is available upon request.

## Additional files

Additional file 1
**Supplementary Information.**

Additional file 2
**MATLAB code of (i) the design approach and (ii) chlorophyll fluorescence induction model and corresponding data.**
