# Supplementary material for: Rational selection of experimental readout and intervention sites for reducing uncertainties in computational model predictions
Source: BMC Bioinformatics. 2015 Jan 16;16:13. doi: 10.1186/s12859-014-0436-5 (PMC4310145; doi:10.1186/s12859-014-0436-5)
Supplement: Additional file 1 — Supplementary Information. [file 12859_2014_436_MOESM1_ESM.pdf]

Supplementary Information

Rational selection of experimental readout and intervention sites for reducing uncertainties in computational model predictions

Robert J Flassig*[1], Iryna Migal[1], Esther van der Zalm[1], Liisa Rihko-Struckmann[1] and Kai Sundmacher[1,2]

[1]Max Planck Institute for Dynamics of Complex Technical Systems, Magdeburg, Germany
[2]Process Systems Engineering Group, Otto von Guericke University, Magdeburg, Germany

January 7, 2015

Contents

1 ***In silico*** **example: model equations** **2**
1.1 Profile likelihood and criterion space for different readout setups 2

2 ***D. salina*** **chlorophyll fluorescence induction model: Criterion space for reducing uncertainties in the model parameters** **3**
2.1 Sensitivity analysis 3
2.2 Classical vs. Profile likelihood based sensitivity indices and entropies . . . 4

*flassig@mpi-magdeburg.mpg.de

1 *In silico* example: model equations

The model equations of the *in silico* example are given as

$$\dot{\mathrm{A}}(t) = u(t) - (k_{11} + k_{21})\mathrm{A}(t) \tag{1}$$

$$\dot{\mathrm{B}}(t) = k_{11}\mathrm{A}(t) - k_{12}\mathrm{B}(t) \tag{2}$$

$$\dot{\mathrm{C}}(t) = k_{12}\mathrm{A}(t) - (k_{22} + k_{23})\mathrm{C}(t) \tag{3}$$

$$\dot{\mathrm{D}}(t) = k_{12}\mathrm{B}(t) + k_{22}\mathrm{C}(t) - d\mathrm{D}(t) \tag{4}$$

with stimulus $u(t) = 1$, states A, B, C, D (which may reflect protein concentrations) and parameters $k_{11}, k_{12}, k_{21}, k_{22}, k_{23}$ and d. We further assumed that $\mathrm{A}(t = 0) \equiv \mathrm{B}(t = 0) \equiv \mathrm{C}(t = 0) \equiv \mathrm{D}(t = 0) \equiv 0$.

1.1 Profile likelihood and criterion space for different readout setups

Here we show the criterion space derived from profile likelihood samples (Figs. S1,S2) .

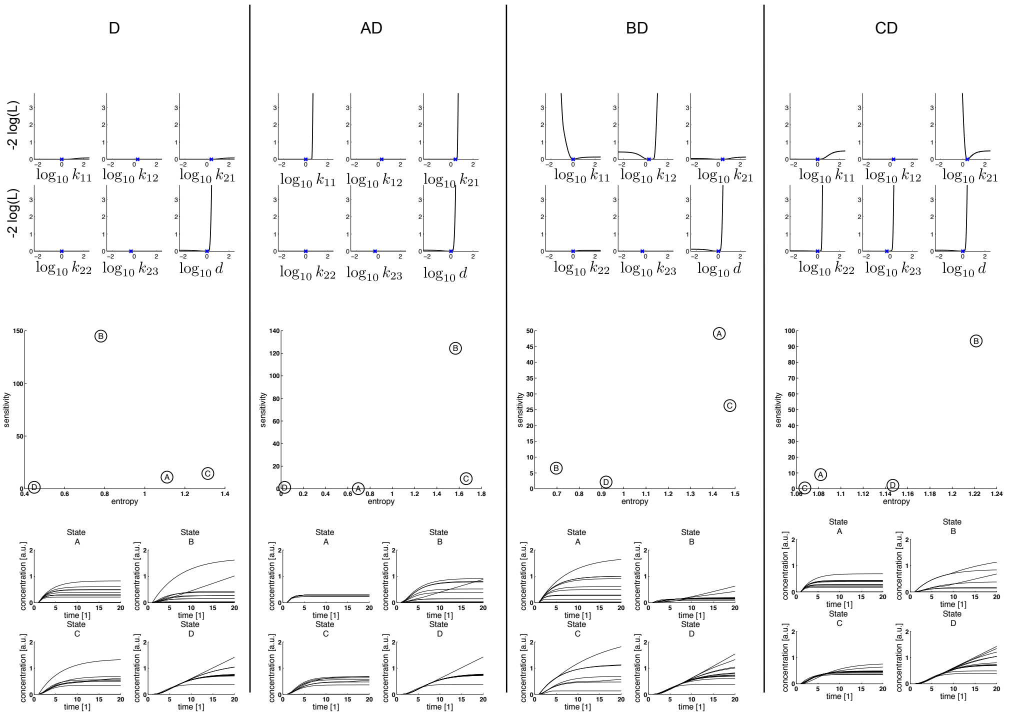

Supplementary Figure S1: Criterion space and profile likelihoods for different readout setups (one and two readouts).

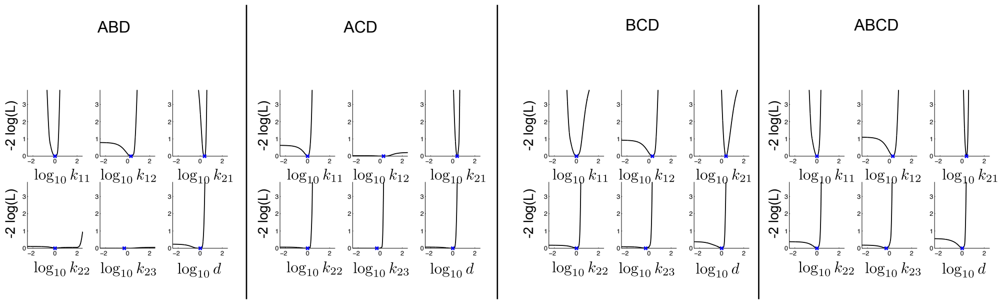

Supplementary Figure S2: Profile likelihoods for different readout setups (three and four readouts).

2 *D. salina* chlorophyll fluorescence induction model: Criterion space for reducing uncertainties in the model parameters

Here we compare profile likelihood based information indices and classical sensitivity indices, which are derived from classical sensitivity analysis (s. Sec. 2.1).

2.1 Sensitivity analysis

In general, sensitivity analysis quantifies how a change in a model parameter influences the model prediction. In the following we restrict the discussion to state trajectory predictions only. As with PLS indices, sensitivity analysis can be used to determine, which predictions or state variables are most affected by parameter uncertainties. For the classical local sensitivity analysis, a system of ordinary differential equations is solved to calculate the sensitivities

$$\frac{d\mathbf{S}}{dt} = \mathbf{JS} + \mathbf{F}_\theta, \tag{5}$$

where $\mathbf{S} = \frac{dx_j}{d\theta_i}$ denotes the sensitivity matrix, $\mathbf{J} = \frac{df_j}{dx_j}$ is the Jacobian of the given system and $\mathbf{F}_\theta = \frac{df_j}{d\theta_i}$. x_j indicates the state variable. To be able to compare all sensitivity values s_{ij} it is useful to normalize them. To ensure the positivity of the results the sensitivities are also squared

$$\bar{s}_{ijk} = \left(s_{ij}(t_k) \frac{\theta_i}{x_i(t_k)} \right)^2 . \tag{6}$$

These coefficients should qualitatively correspond to the PLS indices, although nonlinearity features of the model and parameter interdependencies are neglected. Further, we may as well calculate classical PLS entropy, by using $\bar{s}_{ijk}$ instead of the profile likelihood based s_{ijk} indices. Note that in the main text we introduce the PLS index of parameter

θ_i at time point t_k for *one* specific prediction as s_{ik}. Here the prediction refers to the set of unmeasured model states j (= *a set of predictions*), which is the reason for introducing the third subscript-index j.

2.2 Classical vs. Profile likelihood based sensitivity indices and entropies

The relation between classical $\bar{s}_{ijk}$ and profile likelihood based sensitivity indices s_{ijk} is depicted in Fig. S3 for the model of *D. salina*. We see that classical based indices show qualitative differences compared to profile likelihood based ones (Fig. S4).
In Fig. S5, the criterion space is depicted for classical and profile likelihood based sensitivity indices. When only looking at the sensitivity indices, the classical based approach favorsPQ, Q_B^-, Q_B^{2-} and $A^\star$, in contrast to profile likelihood based predictions, which only favor Q_B^- and Q_B^{2-}. Based on the profile likelihood criterion space, one would favor PQ for an additional readout with regard to equal effects on uncertainty reduction and distribution along the parameters. Classical based criteria would favor A^* and Q_B^{2-}.
In Fig. 4 and also in Tab. 4 of the main document, the performance on PQ and Q_B^- is illustrated in terms of the profile likelihood.

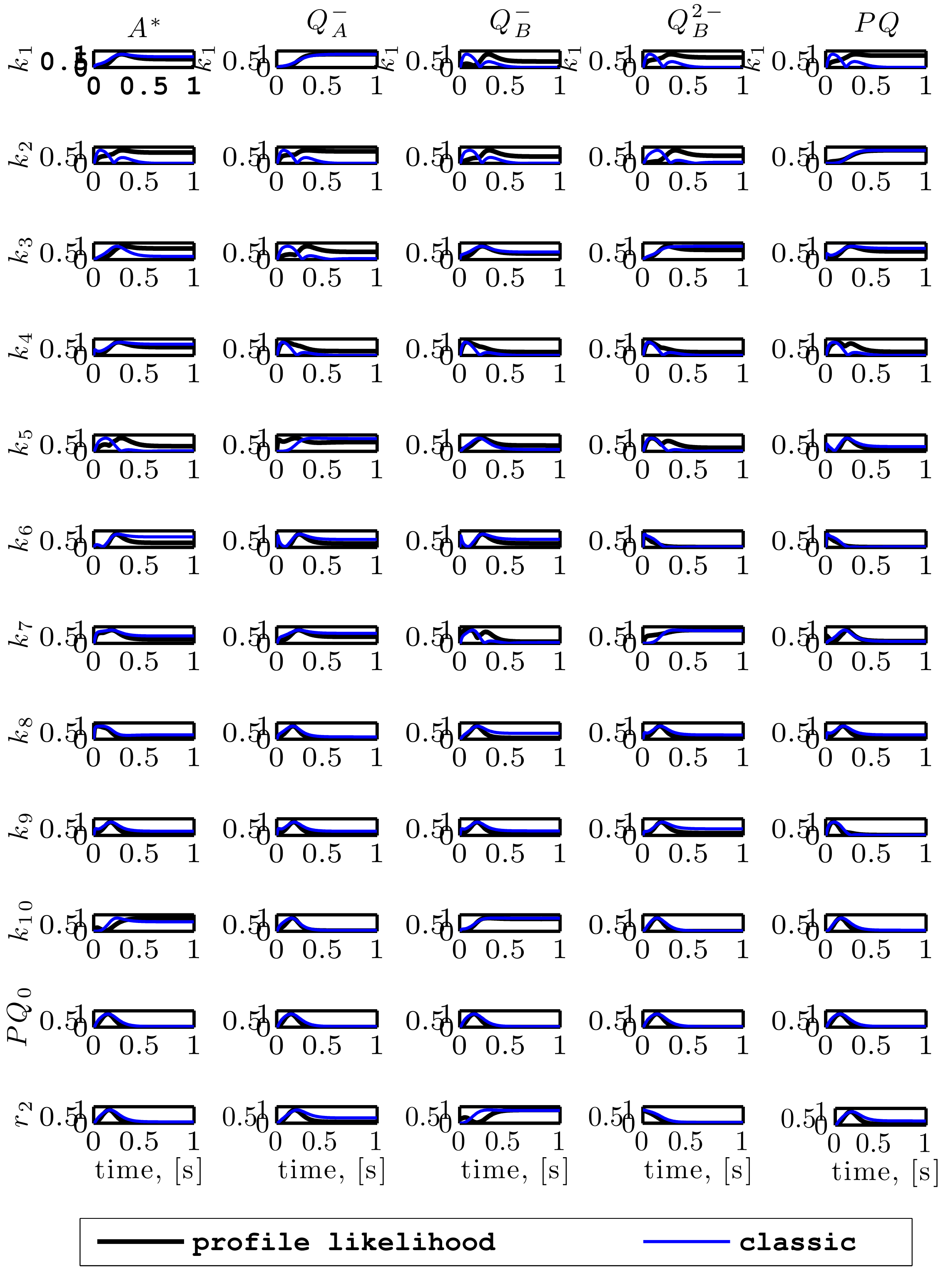

Supplementary Figure S3: Comparison of sensitivity indices for all model states and parameters. Sensitivity indices based on classical, local sensitivity equtions (blue) and profile likelihood samples (black) were calculated and scaled for qualitative comparison.

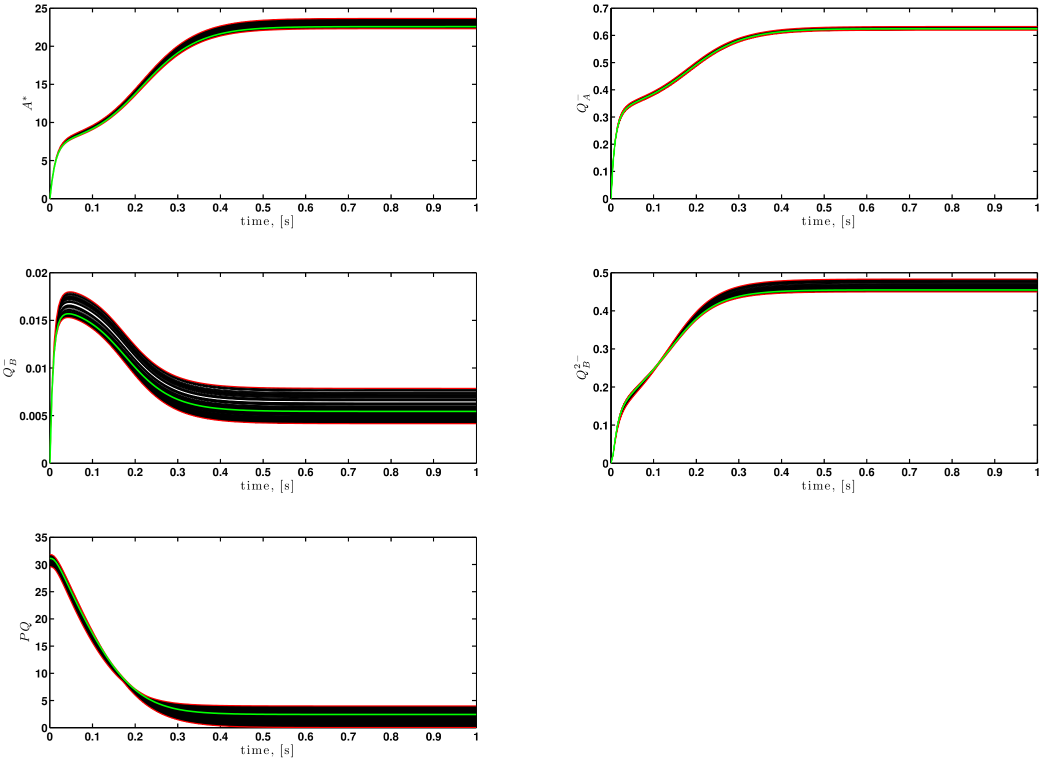

Supplementary Figure S4: Trajectories of internal model states along the profile likelihood of each parameter. Calculated time profiles for all parameter sets along the profile likelihood of each parameter are presented in black. Red curves indicate the lower and upper bounds of the trajectories. Green curves represent the model simulation with the optimal parameter set.

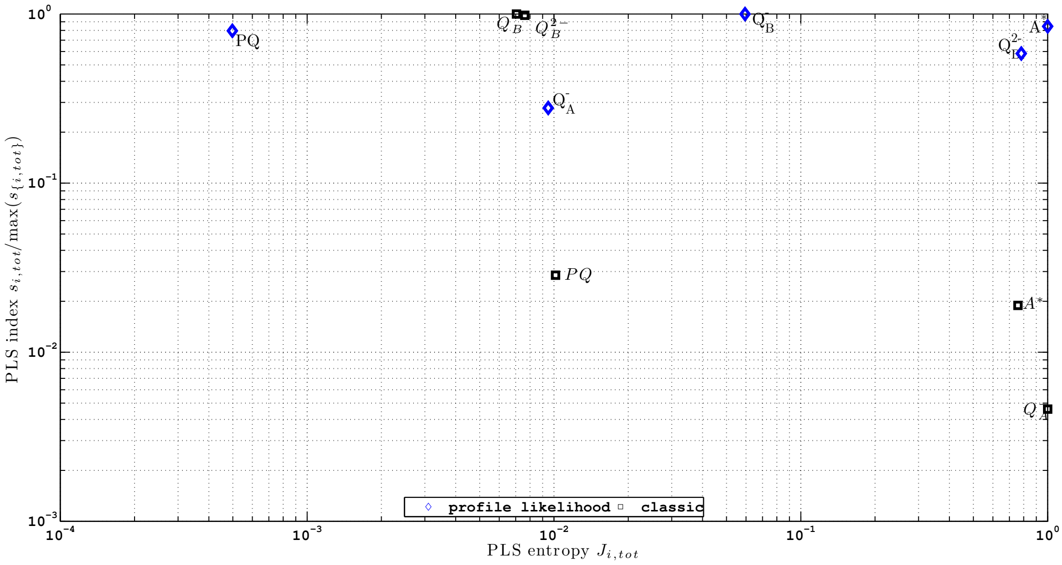

Supplementary Figure S5: Parameter contribution to the prediction sensitivity index. Normalized sensitivity indices are plotted versus the sensitivity entropy. The analysis was performed based on classical, local sensitivity equations (blue) as well as on profile likelihood (black). Index i refers to the states.
