# Supplementary material for: Rational selection of experimental readout and intervention sites for reducing uncertainties in computational model predictions
Source: BMC Bioinformatics. 2015 Jan 16;16:13. doi: 10.1186/s12859-014-0436-5 (PMC4310145; doi:10.1186/s12859-014-0436-5)
Supplement: Additional file 2 — MATLAB code of (i) the design approach and (ii) chlorophyll fluorescence induction model and corresponding data. [file 12859_2014_436_MOESM2_ESM.zip › software/fluorescence_model/readmefirst.rtf]

author: robert j flassiglast update: october-18-2014contact: flassig@mpi-magdeburg.mpg.dethis folder contains supplementary material for chlorophyll fluorescence induction model.simply execute simulate_data_with_model.mto see the performance on the data set.we provide further data for the light intensitiesA.mat --> 95B.mat --> 126(C.mat --> 166)D.mat --> 273E.mat --> 1028in units {\mu}E{\cdot}m^{-2}s^{-1}
