# Supplementary material for: Rational selection of experimental readout and intervention sites for reducing uncertainties in computational model predictions
Source: BMC Bioinformatics. 2015 Jan 16;16:13. doi: 10.1186/s12859-014-0436-5 (PMC4310145; doi:10.1186/s12859-014-0436-5)
Supplement: Additional file 2 — MATLAB code of (i) the design approach and (ii) chlorophyll fluorescence induction model and corresponding data. [file 12859_2014_436_MOESM2_ESM.zip › software/insilico_oed/readmefirst.rtf]

author: robert j flassiglast update: august-18-2014contact: flassig@mpi-magdeburg.mpg.dethis folder contains supplementary material for- calculating profile likelihood given in silico data- evalutating the current identifiability/uncertainty situation to select (i) new readouts and/or (ii) inhibition sites===   before you start ====the profile likelihood calculation uses compiled model functions. here i provide compiled models for linux and mac. other machine compilations are available upon request (windows 32 or 64 bit). you may also compile the model (ode equations) by yourself usinginsilico.txtwhich contains the in silico example in ode form for the systems biology toolbox from henning schmidt:http://www.sbtoolbox2.org/main.phpafter you installed the toolbox (very easy, follow the instruction on the webpage) simply executeoldFolder = cd('../SBTOOLBOX2/')installSB('quick')cd(oldFolder)oldFolder = cd('../SBPD/')installSBPD('quick')cd(oldFolder)%model = SBmodel('insilico.txt');SBPDmakeMEXmodel(model);to compile  the model for your machine architecture. the compiled model needs to be place in the folder of this readme file. also note, the paths to SBTOOLBOX2 and SBPD need to be adjusted accordingly in the above code snippet.==== analysis ====--> if you want to see, how the optimal experimental design works, look (!) at and execute- simulate_along_pl.m for additional readout selection- simulate_along_pl_KO.m for inhibition site identification--> the profile likelihood samples of the parameters for given experimental (in silico) settings, can be generated with- pla_script.m (wrapper)- pl_script_para_model.mwhereas you also have to look into the code to see, how for instance to account for inhibition--> finally, the profile likelihood alone is compute in here - ple.m
