# Supplementary material for: Rational selection of experimental readout and intervention sites for reducing uncertainties in computational model predictions
Source: BMC Bioinformatics. 2015 Jan 16;16:13. doi: 10.1186/s12859-014-0436-5 (PMC4310145; doi:10.1186/s12859-014-0436-5)
Supplement: Additional file 2 — MATLAB code of (i) the design approach and (ii) chlorophyll fluorescence induction model and corresponding data. [file 12859_2014_436_MOESM2_ESM.zip › software/readmefirst.rtf]

author: robert j flassiglast update: october-18-2014contact: flassig@mpi-magdeburg.mpg.dethis folder contains two subfolders- insilico_oed--> here you may play around with the OED strategy (i) selecting new readouts, (ii) selecting inhibition sites--> in the folder, there is a seperate readmefirst file. start there first, after you have finished reading the following lines:--> you can also have a look at the profile likelihood estimation--> the profile likelhoods for the scenarios presented in Flassig et al. 2014, submitted, BMC Bioinformatics are already given. the naming conventions arepla_x123_param_data_1.mathere, data for y={y1,y2,y3,y4} have been given, and this file contains profile likelhood informations on parameter 1.additionally,pla_x44_pko_1_param_data_6.matmeans, y={y1,y2,y3,y4} and additional measurement of y=y4 with inhibition of parameter 1%1 y={1,4} %2 y={2,4} %3 y={3,4} %4 y={4} %initial %12 y={1,2,4} %13 y={1,3,4} %23 y={2,3,4} %123 y={1,2,3,4} %1230 y={1,2,3,4} and additional measurement signal of the KO data in y={4} %1232 y={1,2,3,4} and additional measurement signal of the KO data in y={2} %1233 y={1,2,3,4} and additional measurement signal of the KO data in y={3} %note, KO=KNOCKOUT=INHIBTION, and ist to set in pl_script_para_model- fluorescence_modelin this folder you will find-->  a seperate readmefirst file. start there first, after you have finished reading the following lines:- data of the fluorescence measurement- simulation of the data
